# (*N*-Benzyl-*N*-ethyl­dithio­carbamato)di-*tert*-butyl­chloridotin(IV)

**DOI:** 10.1107/S1600536811006398

**Published:** 2011-02-26

**Authors:** Amirah Faizah Abdul Muthalib, Ibrahim Baba, Mohamed Ibrahim Mohamed Tahir, Edward R. T. Tiekink

**Affiliations:** a School of Chemical Sciences and Food Technology, Faculty of Science and Technology, Universiti Kebangbaan Malaysia, 43600 Bangi, Malaysia; b Department of Chemistry, Universiti Putra Malaysia, 43400 Serdang, Malaysia; c Department of Chemistry, University of Malaya, 50603 Kuala Lumpur, Malaysia

## Abstract

The Sn^IV^ atom in the title diorganotin dithio­carbamate, [Sn(C_4_H_9_)_2_Cl(C_10_H_12_NS_2_)], is penta­coordinated by an asymmetrically coordinating dithio­carbamate ligand, a Cl and two C atoms of the Sn-bound *tert*-butyl groups. The resulting C_2_ClS_2_ donor set defines a coordination geometry inter­mediate between square pyramidal and trigonal bipyramidal with a slight tendency towards the former. In the crystal structure, C—H⋯π contacts link centrosymmetrically related mol­ecules into dimeric aggregates.

## Related literature

For a review on the applications and structural chemistry of tin dithio­carbamates, see: Tiekink (2008 ▶). For additional structural analysis, see: Addison *et al.* (1984 ▶); Spek (2009 ▶). For a recently reported related structure, see: Abdul Muthalib *et al.* (2010 ▶).
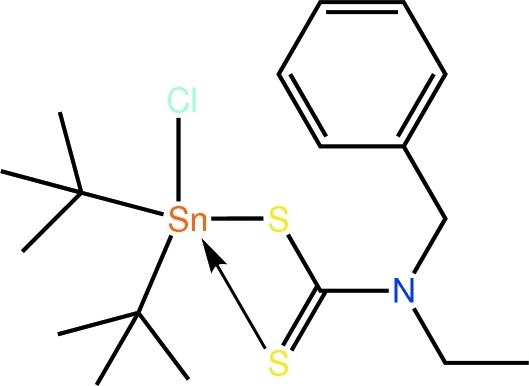


## Experimental

### 

#### Crystal data

[Sn(C_4_H_9_)_2_Cl(C_10_H_12_NS_2_)]
*M* *_r_* = 478.69
Triclinic, 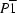

*a* = 8.6140 (2) Å
*b* = 10.9604 (3) Å
*c* = 11.4765 (3) Å
α = 91.858 (2)°
β = 96.193 (2)°
γ = 96.011 (2)°
*V* = 1070.24 (5) Å^3^
*Z* = 2
Mo *K*α radiation
μ = 1.51 mm^−1^
*T* = 150 K
0.30 × 0.23 × 0.16 mm

#### Data collection

Oxford Diffraction Xcaliber Eos Gemini diffractometer
Absorption correction: multi-scan (*CrysAlis PRO*; Oxford Diffraction, 2010 ▶) *T*_min_ = 0.935, *T*_max_ = 1.000
26998 measured reflections
4865 independent reflections
4707 reflections with *I* > 2σ(*I*)
*R*_int_ = 0.034

#### Refinement

*R*[*F* ^2^ > 2σ(*F* ^2^)] = 0.018
*wR*(*F* ^2^) = 0.046
*S* = 1.11
4865 reflections
215 parameters
H-atom parameters constrained
Δρ_max_ = 0.29 e Å^−3^
Δρ_min_ = −0.50 e Å^−3^

### 

Data collection: *CrysAlis PRO* (Oxford Diffraction, 2010 ▶); cell refinement: *CrysAlis PRO*; data reduction: *CrysAlis PRO*; program(s) used to solve structure: *SHELXS97* (Sheldrick, 2008 ▶); program(s) used to refine structure: *SHELXL97* (Sheldrick, 2008 ▶); molecular graphics: *ORTEP-3* (Farrugia, 1997 ▶) and *DIAMOND* (Brandenburg, 2006 ▶); software used to prepare material for publication: *publCIF* (Westrip, 2010 ▶).

## Supplementary Material

Crystal structure: contains datablocks global, I. DOI: 10.1107/S1600536811006398/zs2099sup1.cif

Structure factors: contains datablocks I. DOI: 10.1107/S1600536811006398/zs2099Isup2.hkl

Additional supplementary materials:  crystallographic information; 3D view; checkCIF report

## Figures and Tables

**Table 1:** Selected bond lengths (Å)

Sn—Cl1	2.4847 (4)
Sn—S1	2.4760 (4)
Sn—S2	2.7409 (4)
Sn—C11	2.1884 (14)
Sn—C15	2.1879 (15)

**Table 2:** Hydrogen-bond geometry (Å, °) *Cg*1 is the centroid of the C5–C10 ring.

*D*—H⋯*A*	*D*—H	H⋯*A*	*D*⋯*A*	*D*—H⋯*A*
C3—H3a⋯*Cg*1	0.98	2.78	3.6491 (18)	149
C13—H13b⋯*Cg*1^i^	0.98	2.96	3.5401 (18)	119

Symmetry code: (i) 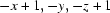.

**Fig. 1.**
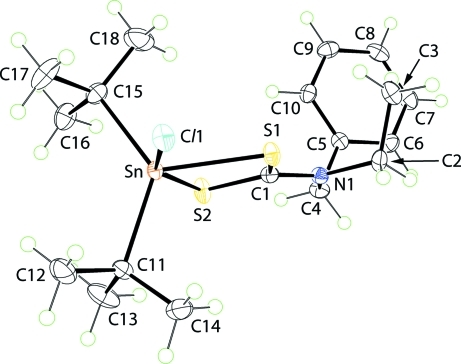
The molecular structure of of (I) showing the atom-labelling scheme and displacement ellipsoids at the 50% probability level.

**Fig. 2.**
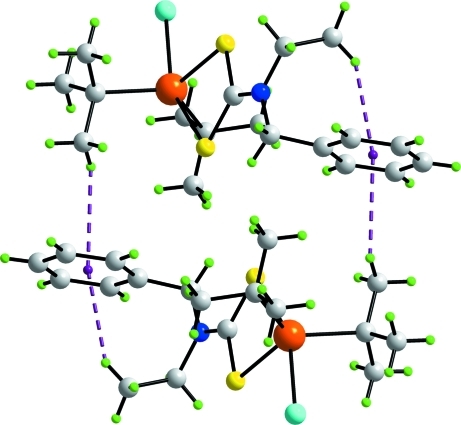
A view of the dimeric aggregate in (I) showing the intra- and intermolecular C–H···π contacts as purple dashed lines.

**Fig. 3.**
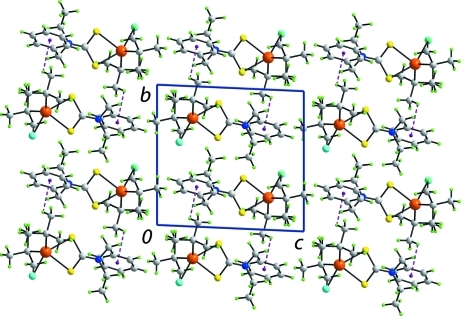
A view in projection down the a axis of (I) showing columns of dimeric aggregates along a. The intermolecular C–H···π contacts are shown as purple dashed lines.

## supplementary crystallographic information

### Comment

Organotin dithiocarbamates attract attention as they exhibit properties suggesting their potential as anti-cancer agents, anti-microbials and insecticides (Tiekink, 2008). Motivated by these and in continuation of structural studies of these systems (Abdul Muthalib *et al.*, 2010), the analysis of the title compound, (I), was undertaken.

The Sn^IV^ atom in (I) is five-coordinated, being chelated by an asymmetrically coordinating dithiocarbamate ligand, a Cl and two C atoms of the Sn-bound *tert*-butyl groups (Fig. 1 and Table 1). The asymmetric chelating mode of the non-symmetric dithiocarbamate ligand is reflected in the non-equivalence of the associated C≐S bond distances (Table 1). The coordination geometry is intermediate between square pyramidal and trigonal bi-pyramidal with a leaning towards the former. This assignment is based on the value calculated for τ of 0.45 for the Sn atom, which compares to the τ values of 0.0 and 1.0 for ideal square pyramidal and trigonal bi-pyramidal geometries, respectively (Spek, 2009; Addison *et al.*, 1984). The mode of coordination of the dithiocarbamate ligand, the disposition of the ligand donor set, and the intermediate coordination geometry observed for (I) matches with the literature precedents (Tiekink, 2008).

The most prominent feature of the crystal packing is the presence of C–H···π interactions (Table 2). As shown in Fig. 2, these lead to dimeric aggregates. It is also noted that intramolecular C–H···π contacts are present so that the benzene ring participates in two such interactions (Table 2, Fig. 2). The dimeric aggregates stack into columns along the *a* axis (Fig. 3).

### Experimental

The dithiocarbamate ligand was prepared by the addition of carbon disulfide (0.01 mol) to an ethanolic solution (20 ml) of ethylbenzylamine (0.01 mol). The mixture was stirred for 1 h at 277 K, after which the solution was added drop wise to a solution of di-*tert*-butyltin(IV) dichloride (0.005 mol) in ethanol (20 ml). The resulting mixture was stirred for 1 h. The white precipitate was filtered, washed with cold ethanol and dried in a desiccator. Crystallization was carried out by using an ethanol:chloroform (1:2) mixture. Yield 76%; m.p. 451–453 K. Elemental analysis. Found (calculated) for C_18_H_30_ClNS_2_Sn: C, 44.81 (45.16); H 6.27 (6.32), N 2.72 (2.93), S 13.23 (13.40); Sn 23.98 (24.80) %. UV (CHCl_3_) λ_max_ 244 (*L*(π) →*L*(π*)). IR (KBr): ν(C—H) 2933*m*, 2958*m*; ν(C≐N) 1496*m*; ν(N—C) 1185 s; ν(C≐S) 950 s; ν(Sn—S) 351 s cm^-1^.

### Refinement

Carbon-bound H-atoms were placed in calculated positions (C—H = 0.95 to 0.99 Å) and were included in the refinement in the riding model approximation, with *U*_iso_(H) set to 1.2 to 1.5*U*_eq_(C).

### Crystal data

**Table:**

[Sn(C_4_H_9_)_2_Cl(C_10_H_12_NS_2_)]	*Z* = 2
*M**_r_* = 478.69	*F*(000) = 488
Triclinic, *P*1	*D*_x_ = 1.485 Mg m^−^^3^
Hall symbol: -P 1	Melting point = 451–453 K
*a* = 8.6140 (2) Å	Mo *K*α radiation, λ = 0.71073 Å
*b* = 10.9604 (3) Å	Cell parameters from 22888 reflections
*c* = 11.4765 (3) Å	θ = 2.4–28.8°
α = 91.858 (2)°	µ = 1.51 mm^−^^1^
β = 96.193 (2)°	*T* = 150 K
γ = 96.011 (2)°	Block, colourless
*V* = 1070.24 (5) Å^3^	0.30 × 0.23 × 0.16 mm

### Data collection

**Table:**

Oxford Diffraction Xcaliber Eos Gemini diffractometer	4865 independent reflections
Radiation source: fine-focus sealed tube	4707 reflections with *I* > 2σ(*I*)
graphite	*R*_int_ = 0.034
Detector resolution: 16.1952 pixels mm^-1^	θ_max_ = 27.5°, θ_min_ = 2.4°
ω scans	*h* = −11→11
Absorption correction: multi-scan (*CrysAlis PRO*; Oxford Diffraction, 2010)	*k* = −13→13
*T*_min_ = 0.935, *T*_max_ = 1.000	*l* = −14→14
26998 measured reflections	

### Refinement

**Table:**

Refinement on *F*^2^	Primary atom site location: structure-invariant direct methods
Least-squares matrix: full	Secondary atom site location: difference Fourier map
*R*[*F*^2^ > 2σ(*F*^2^)] = 0.018	Hydrogen site location: inferred from neighbouring sites
*wR*(*F*^2^) = 0.046	H-atom parameters constrained
*S* = 1.11	*w* = 1/[σ^2^(*F*_o_^2^) + (0.0236*P*)^2^ + 0.279*P*] where *P* = (*F*_o_^2^ + 2*F*_c_^2^)/3
4865 reflections	(Δ/σ)_max_ = 0.003
215 parameters	Δρ_max_ = 0.29 e Å^−^^3^
0 restraints	Δρ_min_ = −0.50 e Å^−^^3^

### Special details

**Table:**

Geometry. All s.u.'s (except the s.u. in the dihedral angle between two l.s. planes) are estimated using the full covariance matrix. The cell s.u.'s are taken into account individually in the estimation of s.u.'s in distances, angles and torsion angles; correlations between s.u.'s in cell parameters are only used when they are defined by crystal symmetry. An approximate (isotropic) treatment of cell s.u.'s is used for estimating s.u.'s involving l.s. planes.
Refinement. Refinement of *F*^2^ against ALL reflections. The weighted *R*-factor *wR* and goodness of fit *S* are based on *F*^2^, conventional *R*-factors *R* are based on *F*, with *F* set to zero for negative *F*^2^. The threshold expression of *F*^2^ > 2σ(*F*^2^) is used only for calculating *R*-factors(gt) *etc*. and is not relevant to the choice of reflections for refinement. *R*-factors based on *F*^2^ are statistically about twice as large as those based on *F*, and *R*- factors based on ALL data will be even larger.

### Fractional atomic coordinates and isotropic or equivalent isotropic displacement parameters (Å^2^)

**Table:**

	*x*	*y*	*z*	*U*_iso_*/*U*_eq_	
Sn	0.908142 (10)	0.227855 (8)	0.757926 (8)	0.01355 (4)	
Cl1	1.09329 (4)	0.40294 (3)	0.84821 (3)	0.02158 (8)	
S1	0.88193 (4)	0.35904 (3)	0.58681 (3)	0.01801 (8)	
S2	0.70139 (5)	0.11432 (3)	0.57914 (3)	0.02180 (8)	
N1	0.69363 (14)	0.26534 (11)	0.39968 (10)	0.0152 (2)	
C1	0.74996 (17)	0.24573 (13)	0.50861 (12)	0.0154 (3)	
C2	0.73841 (18)	0.38150 (14)	0.34275 (13)	0.0192 (3)	
H2A	0.7233	0.3660	0.2566	0.023*	
H2B	0.8514	0.4073	0.3659	0.023*	
C3	0.6448 (2)	0.48595 (15)	0.37411 (15)	0.0248 (3)	
H3A	0.5338	0.4643	0.3450	0.037*	
H3B	0.6849	0.5609	0.3380	0.037*	
H3C	0.6555	0.4998	0.4595	0.037*	
C4	0.58006 (18)	0.17438 (14)	0.32856 (13)	0.0185 (3)	
H4A	0.5613	0.0996	0.3734	0.022*	
H4B	0.6243	0.1508	0.2560	0.022*	
C5	0.42662 (17)	0.22668 (13)	0.29687 (13)	0.0162 (3)	
C6	0.38063 (18)	0.25643 (15)	0.18239 (13)	0.0206 (3)	
H6	0.4422	0.2374	0.1217	0.025*	
C7	0.24535 (19)	0.31377 (15)	0.15584 (14)	0.0248 (3)	
H7	0.2146	0.3341	0.0775	0.030*	
C8	0.15570 (19)	0.34106 (14)	0.24459 (15)	0.0244 (3)	
H8	0.0644	0.3818	0.2273	0.029*	
C9	0.19895 (18)	0.30901 (15)	0.35845 (15)	0.0230 (3)	
H9	0.1362	0.3265	0.4188	0.028*	
C10	0.33337 (17)	0.25166 (14)	0.38442 (13)	0.0185 (3)	
H10	0.3621	0.2293	0.4624	0.022*	
C11	1.08251 (17)	0.09669 (13)	0.75357 (13)	0.0182 (3)	
C12	1.1743 (2)	0.09891 (18)	0.87481 (16)	0.0344 (4)	
H12A	1.2538	0.0413	0.8745	0.052*	
H12B	1.1022	0.0749	0.9326	0.052*	
H12C	1.2258	0.1820	0.8954	0.052*	
C13	1.0023 (2)	−0.03233 (15)	0.7207 (2)	0.0351 (4)	
H13A	0.9457	−0.0337	0.6418	0.053*	
H13B	0.9280	−0.0558	0.7771	0.053*	
H13C	1.0816	−0.0903	0.7221	0.053*	
C14	1.1918 (2)	0.13760 (17)	0.66298 (17)	0.0334 (4)	
H14A	1.2413	0.2211	0.6843	0.050*	
H14B	1.1312	0.1364	0.5855	0.050*	
H14C	1.2731	0.0816	0.6609	0.050*	
C15	0.72963 (18)	0.22899 (15)	0.87978 (13)	0.0210 (3)	
C16	0.6414 (2)	0.10044 (16)	0.87739 (15)	0.0283 (4)	
H16A	0.5648	0.0983	0.9347	0.042*	
H16B	0.7162	0.0408	0.8971	0.042*	
H16C	0.5866	0.0797	0.7988	0.042*	
C17	0.8116 (2)	0.2629 (2)	1.00325 (15)	0.0356 (4)	
H17A	0.8685	0.3453	1.0046	0.053*	
H17B	0.8857	0.2034	1.0253	0.053*	
H17C	0.7330	0.2618	1.0590	0.053*	
C18	0.6177 (2)	0.32236 (19)	0.8403 (2)	0.0390 (5)	
H18A	0.5717	0.3013	0.7594	0.059*	
H18B	0.6756	0.4045	0.8444	0.059*	
H18C	0.5339	0.3212	0.8918	0.059*	

### Atomic displacement parameters (Å^2^)

**Table:**

	*U*^11^	*U*^22^	*U*^33^	*U*^12^	*U*^13^	*U*^23^
Sn	0.01353 (6)	0.01384 (6)	0.01283 (6)	0.00071 (4)	−0.00031 (4)	0.00204 (4)
Cl1	0.02389 (18)	0.01842 (18)	0.01980 (17)	−0.00371 (14)	−0.00377 (14)	0.00095 (13)
S1	0.02071 (18)	0.01593 (17)	0.01529 (16)	−0.00302 (13)	−0.00340 (13)	0.00330 (13)
S2	0.0271 (2)	0.01675 (18)	0.01827 (17)	−0.00533 (15)	−0.00567 (15)	0.00382 (14)
N1	0.0147 (6)	0.0160 (6)	0.0144 (6)	0.0026 (5)	−0.0006 (5)	0.0002 (5)
C1	0.0146 (6)	0.0164 (7)	0.0150 (6)	0.0026 (5)	0.0005 (5)	−0.0010 (5)
C2	0.0198 (7)	0.0232 (8)	0.0148 (7)	0.0010 (6)	0.0023 (6)	0.0058 (6)
C3	0.0268 (8)	0.0179 (8)	0.0292 (8)	0.0013 (6)	0.0012 (7)	0.0057 (6)
C4	0.0196 (7)	0.0188 (7)	0.0160 (7)	0.0042 (6)	−0.0031 (6)	−0.0051 (5)
C5	0.0170 (7)	0.0143 (7)	0.0160 (7)	−0.0001 (5)	−0.0019 (5)	−0.0014 (5)
C6	0.0218 (7)	0.0247 (8)	0.0152 (7)	0.0028 (6)	0.0007 (6)	−0.0009 (6)
C7	0.0262 (8)	0.0254 (8)	0.0210 (8)	0.0038 (6)	−0.0069 (6)	0.0033 (6)
C8	0.0190 (7)	0.0183 (8)	0.0351 (9)	0.0048 (6)	−0.0032 (7)	−0.0005 (7)
C9	0.0199 (7)	0.0220 (8)	0.0267 (8)	−0.0005 (6)	0.0054 (6)	−0.0057 (6)
C10	0.0198 (7)	0.0197 (7)	0.0151 (7)	−0.0009 (6)	0.0008 (6)	−0.0003 (5)
C11	0.0173 (7)	0.0152 (7)	0.0225 (7)	0.0034 (5)	0.0018 (6)	0.0023 (6)
C12	0.0352 (10)	0.0406 (11)	0.0287 (9)	0.0197 (8)	−0.0058 (8)	0.0024 (8)
C13	0.0275 (9)	0.0155 (8)	0.0613 (13)	0.0039 (7)	−0.0013 (9)	0.0013 (8)
C14	0.0362 (10)	0.0296 (10)	0.0402 (10)	0.0125 (8)	0.0202 (8)	0.0069 (8)
C15	0.0190 (7)	0.0236 (8)	0.0206 (7)	−0.0001 (6)	0.0057 (6)	0.0004 (6)
C16	0.0277 (9)	0.0297 (9)	0.0263 (8)	−0.0070 (7)	0.0070 (7)	0.0042 (7)
C17	0.0345 (10)	0.0516 (12)	0.0187 (8)	−0.0090 (8)	0.0097 (7)	−0.0082 (8)
C18	0.0289 (9)	0.0360 (11)	0.0577 (13)	0.0138 (8)	0.0180 (9)	0.0095 (9)

### Geometric parameters (Å, °)

**Table:**

Sn—Cl1	2.4847 (4)	C9—C10	1.384 (2)
Sn—S1	2.4760 (4)	C9—H9	0.9500
Sn—S2	2.7409 (4)	C10—H10	0.9500
Sn—C11	2.1884 (14)	C11—C12	1.522 (2)
Sn—C15	2.1879 (15)	C11—C14	1.523 (2)
S1—C1	1.7470 (15)	C11—C13	1.524 (2)
S2—C1	1.7109 (15)	C12—H12A	0.9800
N1—C1	1.3240 (18)	C12—H12B	0.9800
N1—C4	1.4791 (18)	C12—H12C	0.9800
N1—C2	1.4839 (19)	C13—H13A	0.9800
C2—C3	1.523 (2)	C13—H13B	0.9800
C2—H2A	0.9900	C13—H13C	0.9800
C2—H2B	0.9900	C14—H14A	0.9800
C3—H3A	0.9800	C14—H14B	0.9800
C3—H3B	0.9800	C14—H14C	0.9800
C3—H3C	0.9800	C15—C18	1.525 (2)
C4—C5	1.509 (2)	C15—C16	1.527 (2)
C4—H4A	0.9900	C15—C17	1.530 (2)
C4—H4B	0.9900	C16—H16A	0.9800
C5—C10	1.390 (2)	C16—H16B	0.9800
C5—C6	1.390 (2)	C16—H16C	0.9800
C6—C7	1.391 (2)	C17—H17A	0.9800
C6—H6	0.9500	C17—H17B	0.9800
C7—C8	1.386 (2)	C17—H17C	0.9800
C7—H7	0.9500	C18—H18A	0.9800
C8—C9	1.386 (2)	C18—H18B	0.9800
C8—H8	0.9500	C18—H18C	0.9800
			
C15—Sn—C11	125.79 (6)	C9—C10—C5	120.31 (14)
C15—Sn—S1	117.55 (4)	C9—C10—H10	119.8
C11—Sn—S1	115.59 (4)	C5—C10—H10	119.8
C15—Sn—Cl1	98.77 (4)	C12—C11—C14	110.27 (15)
C11—Sn—Cl1	96.17 (4)	C12—C11—C13	109.80 (14)
S1—Sn—Cl1	84.342 (12)	C14—C11—C13	110.21 (14)
C15—Sn—S2	93.43 (4)	C12—C11—Sn	108.21 (10)
C11—Sn—S2	96.04 (4)	C14—C11—Sn	107.78 (10)
S1—Sn—S2	68.654 (12)	C13—C11—Sn	110.52 (10)
Cl1—Sn—S2	152.989 (12)	C11—C12—H12A	109.5
C1—S1—Sn	91.00 (5)	C11—C12—H12B	109.5
C1—S2—Sn	83.22 (5)	H12A—C12—H12B	109.5
C1—N1—C4	122.17 (12)	C11—C12—H12C	109.5
C1—N1—C2	121.73 (12)	H12A—C12—H12C	109.5
C4—N1—C2	116.09 (11)	H12B—C12—H12C	109.5
N1—C1—S2	123.80 (11)	C11—C13—H13A	109.5
N1—C1—S1	119.10 (11)	C11—C13—H13B	109.5
S2—C1—S1	117.10 (8)	H13A—C13—H13B	109.5
N1—C2—C3	113.75 (12)	C11—C13—H13C	109.5
N1—C2—H2A	108.8	H13A—C13—H13C	109.5
C3—C2—H2A	108.8	H13B—C13—H13C	109.5
N1—C2—H2B	108.8	C11—C14—H14A	109.5
C3—C2—H2B	108.8	C11—C14—H14B	109.5
H2A—C2—H2B	107.7	H14A—C14—H14B	109.5
C2—C3—H3A	109.5	C11—C14—H14C	109.5
C2—C3—H3B	109.5	H14A—C14—H14C	109.5
H3A—C3—H3B	109.5	H14B—C14—H14C	109.5
C2—C3—H3C	109.5	C18—C15—C16	110.55 (15)
H3A—C3—H3C	109.5	C18—C15—C17	111.09 (15)
H3B—C3—H3C	109.5	C16—C15—C17	109.57 (14)
N1—C4—C5	110.67 (12)	C18—C15—Sn	108.38 (11)
N1—C4—H4A	109.5	C16—C15—Sn	108.51 (10)
C5—C4—H4A	109.5	C17—C15—Sn	108.66 (10)
N1—C4—H4B	109.5	C15—C16—H16A	109.5
C5—C4—H4B	109.5	C15—C16—H16B	109.5
H4A—C4—H4B	108.1	H16A—C16—H16B	109.5
C10—C5—C6	119.24 (14)	C15—C16—H16C	109.5
C10—C5—C4	119.60 (13)	H16A—C16—H16C	109.5
C6—C5—C4	121.08 (14)	H16B—C16—H16C	109.5
C5—C6—C7	120.61 (15)	C15—C17—H17A	109.5
C5—C6—H6	119.7	C15—C17—H17B	109.5
C7—C6—H6	119.7	H17A—C17—H17B	109.5
C8—C7—C6	119.51 (15)	C15—C17—H17C	109.5
C8—C7—H7	120.2	H17A—C17—H17C	109.5
C6—C7—H7	120.2	H17B—C17—H17C	109.5
C9—C8—C7	120.14 (15)	C15—C18—H18A	109.5
C9—C8—H8	119.9	C15—C18—H18B	109.5
C7—C8—H8	119.9	H18A—C18—H18B	109.5
C10—C9—C8	120.15 (14)	C15—C18—H18C	109.5
C10—C9—H9	119.9	H18A—C18—H18C	109.5
C8—C9—H9	119.9	H18B—C18—H18C	109.5
			
C15—Sn—S1—C1	−83.40 (7)	C8—C9—C10—C5	0.5 (2)
C11—Sn—S1—C1	85.49 (6)	C6—C5—C10—C9	−2.0 (2)
Cl1—Sn—S1—C1	179.64 (5)	C4—C5—C10—C9	174.62 (14)
S2—Sn—S1—C1	−0.96 (5)	C15—Sn—C11—C12	−55.53 (13)
C15—Sn—S2—C1	119.28 (6)	S1—Sn—C11—C12	136.63 (11)
C11—Sn—S2—C1	−114.17 (6)	Cl1—Sn—C11—C12	49.96 (11)
S1—Sn—S2—C1	0.98 (5)	S2—Sn—C11—C12	−154.18 (11)
Cl1—Sn—S2—C1	2.29 (6)	C15—Sn—C11—C14	−174.77 (11)
C4—N1—C1—S2	0.84 (19)	S1—Sn—C11—C14	17.39 (12)
C2—N1—C1—S2	179.45 (10)	Cl1—Sn—C11—C14	−69.28 (11)
C4—N1—C1—S1	−179.25 (10)	S2—Sn—C11—C14	86.58 (11)
C2—N1—C1—S1	−0.64 (18)	C15—Sn—C11—C13	64.74 (14)
Sn—S2—C1—N1	178.46 (13)	S1—Sn—C11—C13	−103.11 (12)
Sn—S2—C1—S1	−1.46 (7)	Cl1—Sn—C11—C13	170.23 (12)
Sn—S1—C1—N1	−178.32 (11)	S2—Sn—C11—C13	−33.91 (12)
Sn—S1—C1—S2	1.60 (8)	C11—Sn—C15—C18	−171.31 (11)
C1—N1—C2—C3	−82.33 (17)	S1—Sn—C15—C18	−3.68 (13)
C4—N1—C2—C3	96.36 (15)	Cl1—Sn—C15—C18	84.49 (12)
C1—N1—C4—C5	117.52 (15)	S2—Sn—C15—C18	−71.34 (12)
C2—N1—C4—C5	−61.16 (16)	C11—Sn—C15—C16	−51.22 (13)
N1—C4—C5—C10	−67.27 (17)	S1—Sn—C15—C16	116.41 (10)
N1—C4—C5—C6	109.31 (16)	Cl1—Sn—C15—C16	−155.42 (10)
C10—C5—C6—C7	1.8 (2)	S2—Sn—C15—C16	48.75 (11)
C4—C5—C6—C7	−174.75 (14)	C11—Sn—C15—C17	67.86 (14)
C5—C6—C7—C8	−0.2 (2)	S1—Sn—C15—C17	−124.51 (11)
C6—C7—C8—C9	−1.4 (2)	Cl1—Sn—C15—C17	−36.34 (12)
C7—C8—C9—C10	1.2 (2)	S2—Sn—C15—C17	167.83 (12)

### Hydrogen-bond geometry (Å, °)

**Table:**

Cg1 is the centroid of the C5–C10 ring.

**Table:**

*D*—H···*A*	*D*—H	H···*A*	*D*···*A*	*D*—H···*A*
C3—H3a···Cg1	0.98	2.78	3.6491 (18)	149
C13—H13b···Cg1^i^	0.98	2.96	3.5401 (18)	119

Symmetry codes:  (i) −*x*+1, −*y*, −*z*+1.
